# Evidence for conserved DNA and histone H3 methylation reprogramming in mouse, bovine and rabbit zygotes

**DOI:** 10.1186/1756-8935-1-8

**Published:** 2008-11-03

**Authors:** Konstantin Lepikhov, Valeri Zakhartchenko, Ru Hao, Feikun Yang, Christine Wrenzycki, Heiner Niemann, Eckhard Wolf, Joern Walter

**Affiliations:** 1 University of Saarland, Natural Sciences – Technical Faculty III, Biological Sciences, Genetics/Epigenetics, 66123 Saarbrücken, Germany; 2 Department of Molecular Animal Breeding and Biotechnology, Ludwig-Maximilian University, 81377 Munich, Germany; 3 University of Veterinary Medicine, Clinic for Cattle, Reproductive Medicine Unit, 30173 Hannover, Germany; 4 Institute of Animal Breeding (FAL), Department of Biotechnology, Höltystrasse 10, Mariensee, 31535 Neustadt, Germany

## Abstract

**Background:**

In mammals the parental genomes are epigenetically reprogrammed after fertilization. This reprogramming includes a rapid demethylation of the paternal (sperm-derived) chromosomes prior to DNA replication in zygotes. Such active DNA demethylation in the zygote has been documented for several mammalian species, including mouse, rat, pig, human and cow, but questioned to occur in rabbit.

**Results:**

When comparing immunohistochemical patterns of antibodies against 5-methyl-cytosine, H3K4me3 and H3K9me2 modifications we observe similar pronuclear distribution and dynamics in mouse, bovine and rabbit zygotes. In rabbit DNA demethylation of the paternal chromosomes occurs at slightly advanced pronuclear stages. We also show that the rabbit oocyte rapidly demethylates DNA of donor fibroblast after nuclear transfer.

**Conclusion:**

Our data reveal that major events of epigenetic reprogramming during pronuclear maturation, including mechanisms of active DNA demethylation, are apparently conserved among mammalian species.

## Background

DNA methylation in CpG dinucleotides is an important epigenetic signal controlling heterochromatin formation, genomic imprinting, X-inactivation and gene expression [1]. DNA methylation patterns are subject to genome-wide epigenetic reprogramming at certain developmental stages, particularly during certain phases of germ cell and early embryonic development [2]. After fertilization, DNA methylation of sperm and oocyte-derived chromosomes is largely erased. While Southern blot studies of DNA methylation in repetitive elements in mouse gametes suggested that sperm chromosomes are more hypermethylated than those of oocytes [3], a recent analysis using methylated DNA immunoprecipitation revealed equally low DNA methylation levels in both sperm and oocytes, at least in the promoter regions [4]. Hence the observed decrease of DNA methylation during early embryonic development apparently largely reflects demethylation of (some) repetitive elements. Upon further development DNA methylation again increases in cells of the inner cell mass, while cells of the trophectoderm remain rather hypomethylated [5,6]. The dynamics of DNA demethylation during early preimplantation development have been thoroughly investigated by a number of research groups in different mammalian species. Immunohistochemical studies on mouse zygotes using antibodies against 5-methyl-cytosine (α-5meC) showed a rapid loss of DNA methylation exclusively in the paternal pronucleus. The reactivity of the α-5meC antibody starts to diminish around the early pronuclear stage 2 (PN2) when the protamine-histone exchange is completed (approximately three hours post fertilization). At early PN4 (approximately 8 to 10 hours post fertilization) the α-5meC signal is completely absent from the paternal pronucleus [6-8]. Bisulfite sequencing of zygotic DNA confirmed these rapid demethylation events for some single copy sequences and repetitive elements but revealed that imprinting control regions of imprinted genes and certain classes of repeat sequences remain refractory to such general demethylation [9-11].

Pronounced active demethylation of paternal DNA was not only found in mouse but also reported for rat, pig, human and, to a lesser extent, for bovine zygotes [12-15]. It is therefore considered as a general early epigenetic reprogramming event in mammalian development. However, the biological function of this process remains unclear. It has been proposed as being important for early transcriptional control, or as serving as a mechanism to reduce accumulation of transgenerational epigenetic effects propagated through the male germ line [6,7,16-18]. The concept of paternal pronuclear demethylation as a general hallmark of early mammalian development was challenged by reports stating that this process is lacking in rabbit, ovine and pig zygotes [15,19-21]. On the contrary, other experiments demonstrated the capability of mature ovine oocytes to demethylate mouse sperm DNA introduced by intracytoplasmic sperm injection (ICSI) [22]. Moreover, Zhang et al showed a partial loss of DNA methylation at centromeric satellite repeats in rabbit zygotes following ICSI [23]. While these data suggest the existence of DNA demethylation activity in rabbit and ovine oocytes, it remained unclear whether the paternal pronucleus is subject to such demethylation in naturally derived zygotes.

Concomitant with pronuclear DNA methylation reprogramming specific alterations in histone modifications have been observed in early mouse embryos. On the paternal chromosomes protamines are rapidly exchanged by acetylated histones which subsequently become monomethylated at position H3K4 [6,24]. This process coincides with paternal DNA demethylation in the mouse zygote. In addition, particular histone modifications such as di/trimethylation at H3K9, H4K20 and H3K27 are only present on the maternal chromosomes [24-27]. This asymmetry between parental genomes, particular of DNA methylation and H3K9me2, persists until at least the two-cell stage of mouse embryo development [6,24,28,29]. Direct comparative epigenetic studies are still scarce for mammalian species. In our studies we therefore analyzed the dynamics of H3K9me2 and H3K4me3 methylation along with DNA methylation alterations in mouse, bovine and rabbit zygotes.

## Results and discussion

### Dynamics of DNA methylation in mouse, bovine and rabbit zygotes

**Figure 1:**
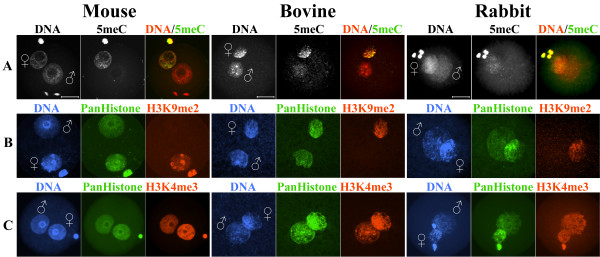
**DNA and histone H3 methylation in mouse, bovine and rabbit zygotes at advanced pronuclear stages**. Representative images (>20 zygotes for each case) of indirect fluorescent immunostaining using specific antibodies against: (A) methylated cytosine residues (5meC); (B) H3K9me2 and (C) H3K4me3. The asymmetry in 5meC and H3K9me2 signal intensities between paternal and maternal pronuclei of mouse, bovine and rabbit zygotes are clearly visible in lanes A and B respectively. H3K4me3-specific signal (lane C) is present in both pronuclei. Scale bar 20 μm.

**Figure 2:**
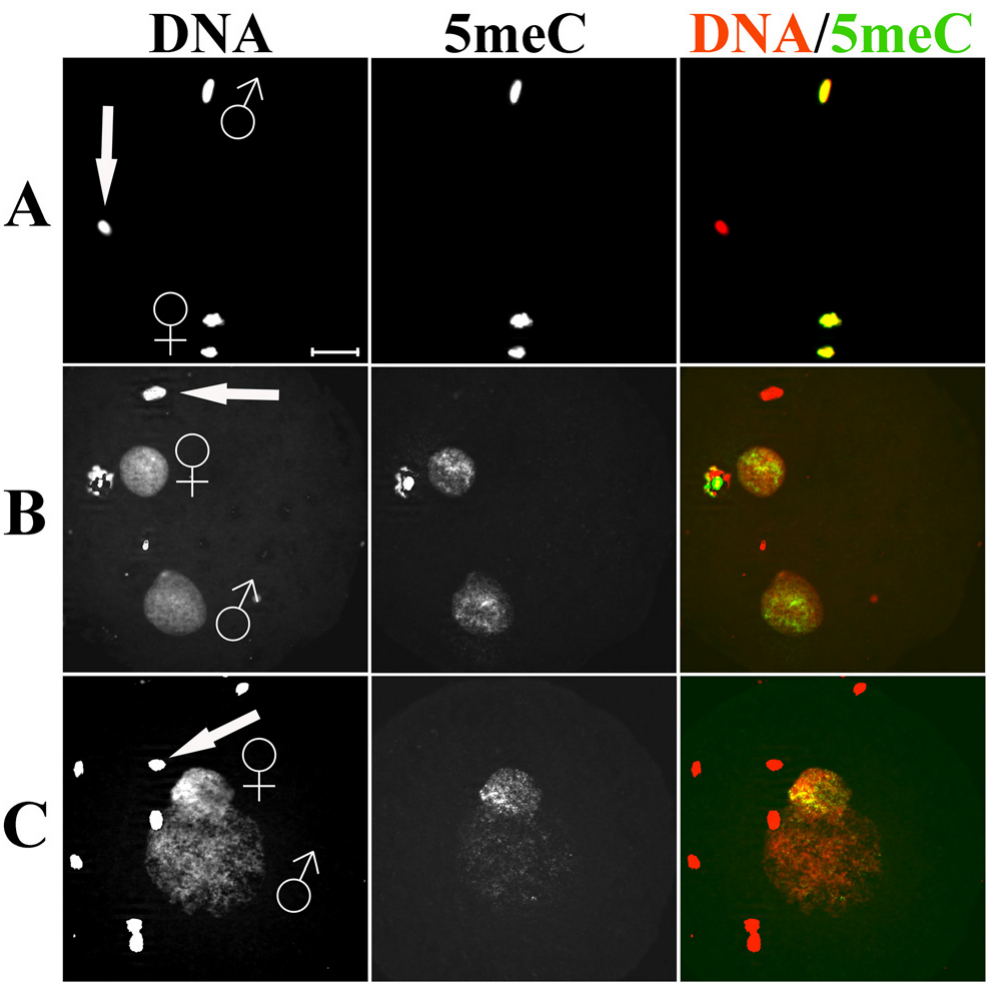
**Anti-5meC immunostaining on rabbit zygotes at various pronuclear stages**. Lane (A): Example of a very early zygote (*n *= 4) (approximately PN0) just after the penetration of sperm into the oocyte. The decondensing sperm chromatin is strongly stained with the α-5meC antibody (in contrast to sperm heads). Two haploid sets of maternal chromosomes are still present in the ooplasm and the second polar body is not yet formed. Lane (B): Example of a zygote (*n *= 3) with newly formed pronuclei (approximately PN2). Both paternal and maternal pronuclei at this stage (approximately 3 to 4 hours after fertilization) show similar α-5meC signal intensities. Lane (C) shows an example of a zygote with expanded pronuclei (approximately PN3-4, 8 to 10 hours post-fertilization, *n *> 20). The α-5meC signal is strongly different between the two parental pronuclei, the paternal chromatin almost devoid of antibody staining. Panels: DNA – images of propidium iodide staining; 5meC – images of α-5meC monoclonal antibody staining; DNA/5meC – merged images. The arrows in the DNA staining panels indicate sperm heads still attached to the zygote. The highly compacted DNA of these sperm heads is not accessible to α-5meC staining in contrast to the just-penetrated sperm shown in (A). Scale bar 20 μm.

**Figure 3:**
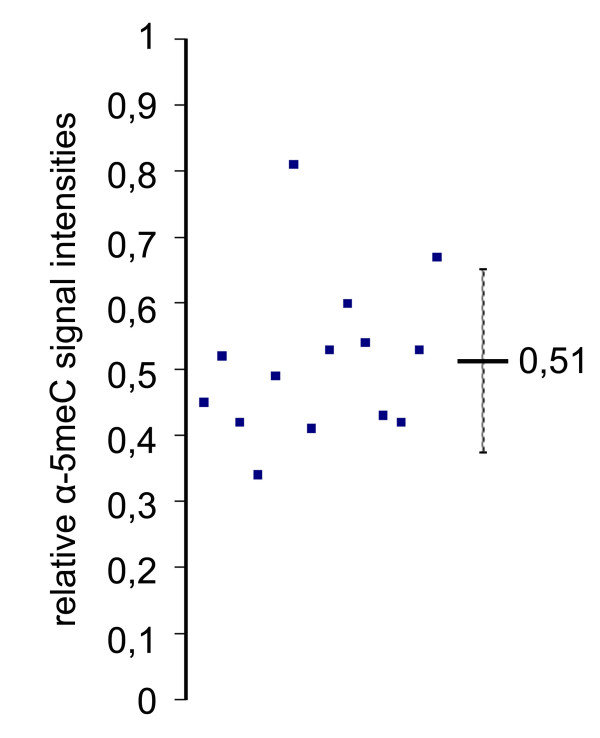
**Quantification of the relative α-5meC signal intensities in paternal pronuclei of rabbit zygotes**. The graph shows the relative α-5meC/DNA paternal to maternal signal intensities ratios for 14 individual PN3-4-stage zygotes. The ratio describes the amount of the remaining paternal methylation compared to maternal (set as 1). The mean value is 0.51, standard deviation is 0.139.

To compare the developmental dynamics of DNA methylation in pronuclei of mouse, rabbit and bovine zygotes we performed indirect immunofluorescence using well-characterized α-5meC-specific monoclonal antibody [30]. Mouse and bovine zygotes were obtained by *in vitro *fertilization. Rabbit zygotes were derived from superovulated females, naturally mated with males. In all three species we found a clear asymmetry of α-5meC staining between the parental pronuclei at advanced stages of zygotic development (more than 6 hours after fertilization). Whereas the DNA of the maternal pronuclei and polar bodies retained a strong reactivity with the antibody, this reactivity was greatly reduced in all paternal pronuclei of the three species (Figure 1A). While the observed loss of DNA methylation signal in paternal DNA in mouse and bovine zygotes is in full agreement with previously published data [6,8], the finding of a strong loss of DNA methylation signal in the paternal pronuclei of rabbit zygotes was unexpected. We therefore carefully re-examined rabbit zygotes at different time points (pronuclear stages) after fertilization. At early stages of development, starting from decondensing sperm and maternal metaphase DNA (PN0-1) up to early expanding stages (PN2) (Figure 2A), both paternal and maternal chromosomes are strongly stained with the α-5meC antibody. In mouse the α-5meC signal is already greatly reduced at the PN2 stage [6]. Upon further expansion of the pronuclei (PN3-4) the α-5meC signal intensity decreases substantially in the paternal but remains unchanged in the maternal pronucleus, respectively (Figure 2C). To validate this visual assessment we quantified the loss of paternal methylation on digital immunofluorescence images of zygotes collected approximately 6 to 8 hours after fertilization. The signal intensities of α-5meC antibody and propidium iodide DNA staining were measured for both paternal and maternal pronuclear areas in individual zygotes and the relative intensity ratios were calculated. Out of approximately 30 PN3-4-staged zygotes obtained, only 14 had non-overlaying pronuclei and hence qualified for ratio determination. In all 14 cases the paternal pronuclei consistently showed a reduction of α-5meC to DNA signal ratio (Figure 3) to an average ratio of 0.51 as compared with matching maternal pronuclei ratio (set as 1). Maternal and paternal pronuclei at earlier stages (PN2, see Figure 2B) exhibited approximately identical intensity ratios (data not shown).

We conclude that in rabbit zygotes the paternal chromosomes undergo a significant demethylation at advanced pronuclear stages. The extent and dynamics of this demethylation is slightly different compared with the mouse. A recent report by Park et al corroborates this interpretation [31]. We can only speculate as to why previous publications came to the different interpretation that a paternal genome-wide DNA demethylation is absent in early rabbit embryos [15,19]. One explanation for this discrepancy may be that the images analyzed in both previous publications show zygotes at rather early developmental stages, that is, at pronuclear stages before we observe DNA demethylation to commence. In addition, the different fixation method used in one of the previous studies may have contributed to the apparently different interpretation of images. We have applied the fixation method used by Shi et al (2004) [19] on mouse zygotes and found it to frequently produce incomplete cellular or nuclear membrane breakdown. In particular we often observed inconsistent pronuclear morphology and highly variable signals in α-5meC staining (data not shown).

### Dynamics of DNA demethylation in rabbit one-cell embryos following somatic nuclear transfer

**Figure 4:**
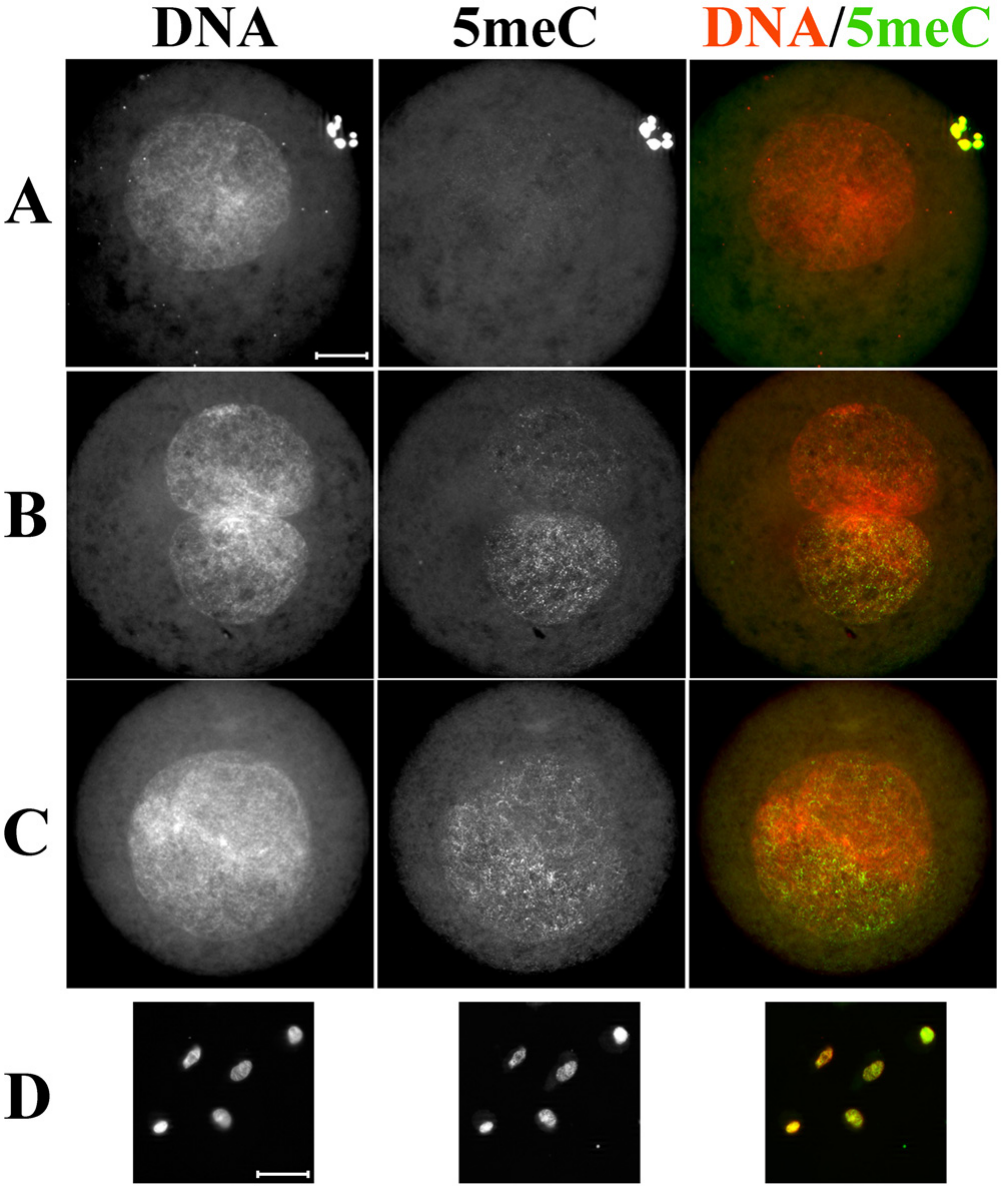
**Anti-5meC immunostaining of rabbit one-cell nuclear transfer embryos**. Rabbit one-cell nuclear transfer (NT) embryos were fixed 4 hours after activation. Immunostaining with α-5meC antibody shows the absence of signal in the majority (A, *n *= 12) of NT embryos, while a partial (B, *n *= 3) or complete (C, *n *= 4) presence of signal is observed less frequently. (D) Control staining on rabbit fetal fibroblasts used as donors for NT. Scale bar 20 μm.

**Figure 5:**
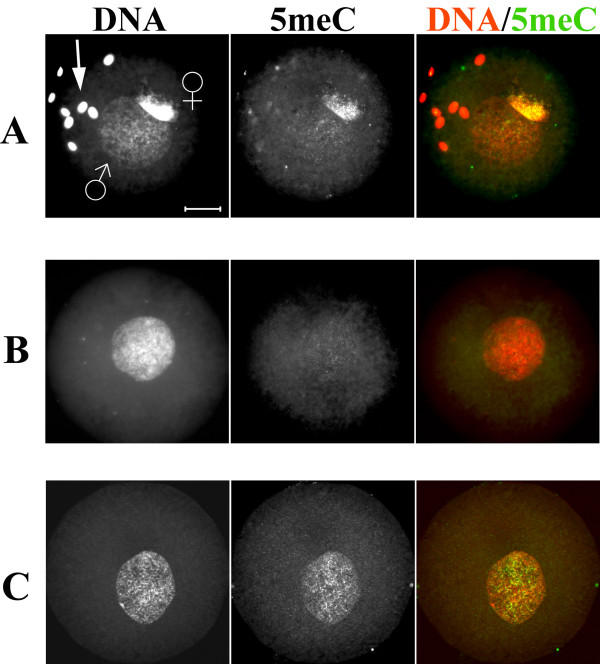
**Anti-5meC immunostaining using a rabbit polyclonal antibody**. The figure shows the efficient staining using a polyclonal antibody, reproducing results shown in Figure 1A and Figure 3. (A) Naturally derived rabbit zygotes and (B and C) rabbit one-cell nuclear transfer (NT) embryos. The arrow points to the sticking sperm head (see Figure 2). (B, *n *= 8) Represents a significant loss of DNA methylation in the NT embryo, (C, *n *= 2) represents a lesser extent of DNA demethylation in NT embryo. Scale bar 20 μm.

To obtain further proof for a demethylation activity in rabbit oocytes we examined one-cell embryos produced by nuclear transfer (NT) using rabbit fetal fibroblasts as nuclear donors. As in naturally fertilized zygotes, we found an already strong reduction of the α-5meC signal in pronuclei of NT embryos at 4 hours after activation (Figure 4A and 4D, respectively). The majority of rabbit NT embryos showed a strong loss of methylation signal in the donor DNA (Figure 4A) and only a few escaped demethylation almost entirely (Figure 4C). Similar variable demethylation was reported for NT-derived bovine embryos [32]. Among NT embryos with only partial demethylation we found a remarkable example in which the transferred single donor nucleus formed two separate pronuclei. Only one of these pronuclei was demethylated at the time of fixation (Figure 4B). For the majority of one-cell NT embryos we estimated at least a four to six-fold decrease of α-5meC signal intensity as measured by DNA/immunofluorescence signal ratios using ImageJ software-based calculations. To exclude that the observed strong reduction of the α-5meC signal in the paternal pronucleus and in NT embryos might be due to selective antibody reactivity and sensitivity we performed a second series of experiments using a different polyclonal α-5meC antibody and obtained identical results (Figure 5A to 5C).

The DNA demethylation in NT embryos is apparently 1 to 2 hours faster (less than 6 hours post-activation) as compared with zygotes (more than 6 hours post-fertilization). The reasons for the 'delayed' demethylation in zygotes remain unclear. It is possible that a completed protamine-histone exchange (1 to 2 hours) is required for demethylation to commence in the zygotes. The chromosomes of donor fibroblast are already in such a histone-containing chromatin state, which allows a more rapid demethylation process. However, it has been shown that also donor nuclei undergo considerable changes upon transfer into enucleated oocytes, including chromosomal decondensation and histone H1 exchange [33].

**Figure 6:**
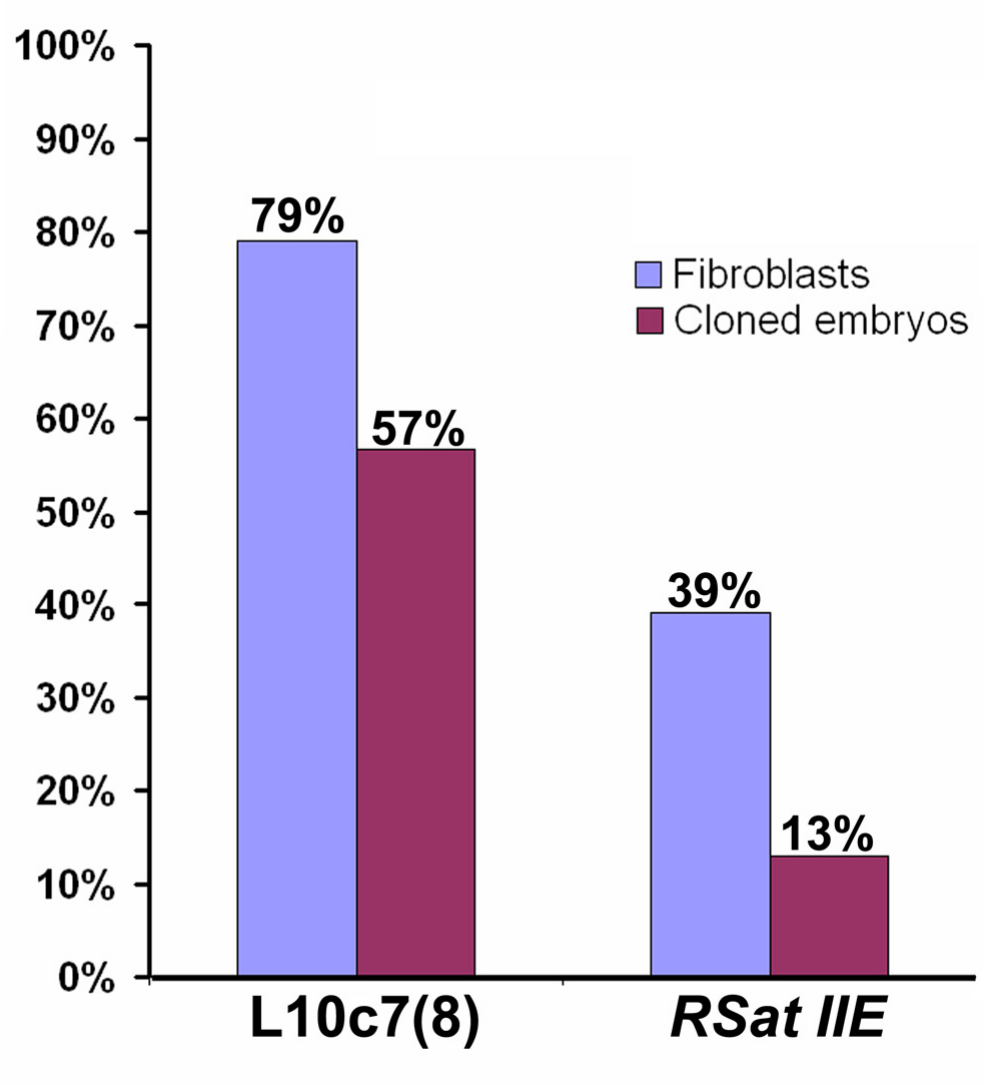
**Bisulfite sequencing data**. Percentage of methylation in CpG dinucleotides in rabbit L1Oc7(8) variants of LINE1 repeated element and satellite repeats *Rsat IIE*. Percentages of methylated CpG sites are calculated in relation to the total number of analysed CpG sites for rabbit fetal fibroblasts and NT embryos.

**Figure 7:**
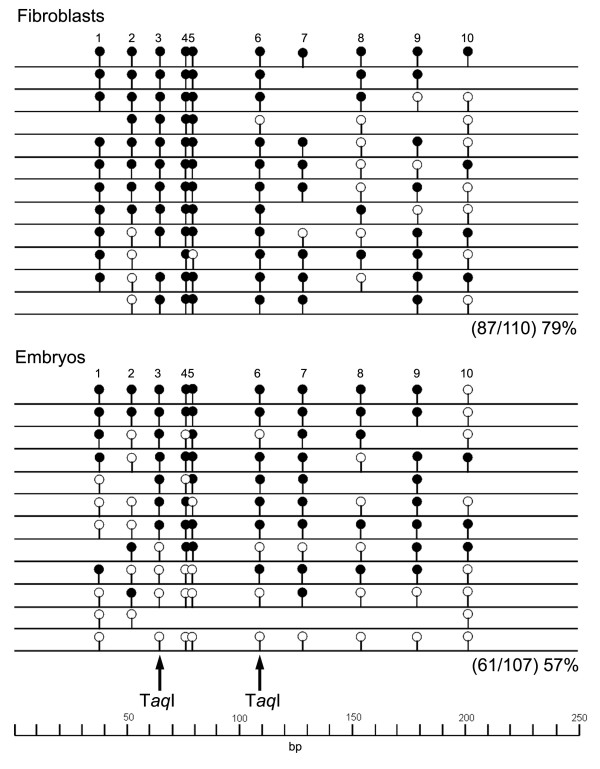
**Bisulfite sequencing profile for L1Oc7(8)**. Graphical representation of individually sequenced bisulfite amplicons highlighting 10 CpG dinucleotide positions analyzed in this LINE1 element class. Open and closed circles indicate unmethylated and methylated CpGs, respectively. Some CpGs are absent in individual clones due sequence polymorphism in L1Oc7(8) copies. T*aq*I sites used for combined bisulfite restriction analysis assays are highlighted by black arrows.

**Figure 8:**
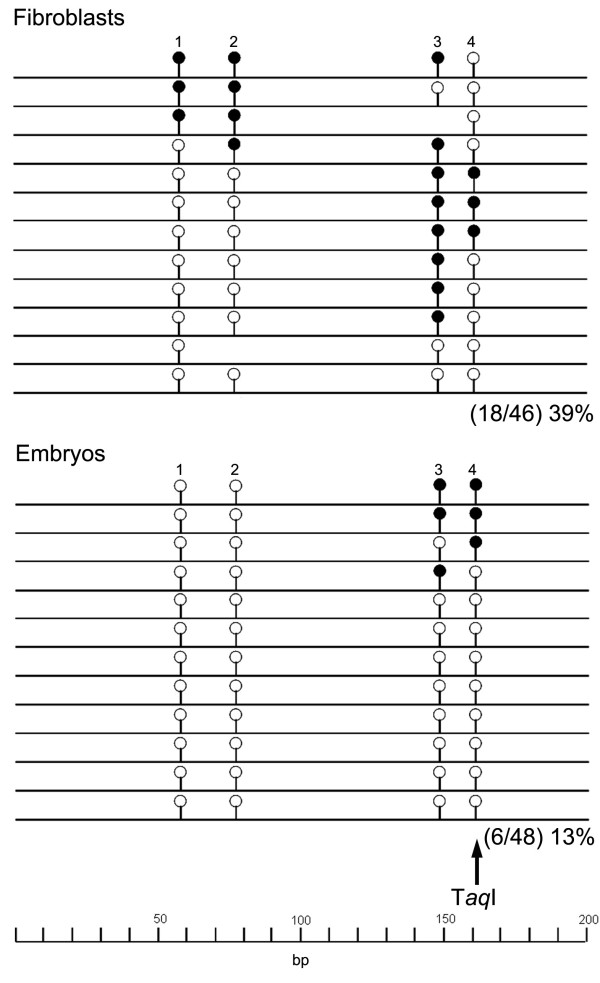
**Bisulfite sequencing profile for *Rsat IIE***. Graphical representation of individually sequenced bisulfite amplicons highlighting four CpG dinucleotide positions analysed in *Rsat IIE *satellite repeat. Open and closed circles indicate unmethylated and methylated CpGs, respectively. T*aq*I site used for combined bisulfite restriction analysis assays are highlighted by black arrows.

To obtain independent proof for the presence of a DNA demethylation activity in the rabbit oocyte we performed comparative bisulfite sequencing analyses with DNA of one-cell NT embryos and fibroblast donor cells, respectively. In particular we examined L1Oc7(8) sequences [34], a variant of LINE1 elements, shown to be partially demethylated in mouse zygotes [11] and *Rsat IIE *satellite sequences [23]. The sequencing data revealed that the overall methylation of L1Oc7(8) decreases from 79% in fibroblasts to 57% in NT embryos and from 39% to 13%, respectively in *Rsat IIE *sequences (Figure 6). Combined bisulfite restriction analysis (COBRA) (see Methods) at two CpG positions within the L1Oc7 amplicons confirmed the sequencing results (data not shown). A detailed analysis of the methylation patterns of L1Oc7(8) and *Rsat IIE *clones (Figures 7 and 8) shows that some CpGs in L1Oc7(8) sequences are more resistant to demethylation than others, and the overall methylation pattern is often mosaic (Figure 7).

The bisulfite data are in agreement with previous data obtained in mouse zygotes where a decrease of about 28% methylation for LINE1 elements was found [11]. However, the extent of demethylation as determined by bisulfite sequencing for repetitive elements is not as pronounced as the significant loss of α-5meC antibody reactivity suggested in immunostainings (Figure 3 and Figure 4). One explanation for this apparent discrepancy might be that a gradual reduction of approximately 25% of co-methylated CpGs within repetitive elements may strongly influence the immunofluorescence detection by α-5meC antibodies. Indeed, Weber et al showed that efficient immunoprecipitation by α-5meC antibodies requires at least five methylated CpGs to be in proximity on the same strand [35].

### H3K9me2 and H3K4me3 in late zygotes and early two-cell embryos of all three species

Immunohistochemical analysis of mouse, bovine and rabbit late-stage zygotes with antibodies against H3K9me2 revealed that this type of histone modification is exclusively associated with maternal pronuclei in all three species (Figure 1B). We conclude that not only the pronuclear asymmetry of DNA methylation but also the asymmetry of H3K9me2 appears to be ubiquitous for late-stage zygotes of different mammalian species. Given that the maternal pronuclear DNA is refractory to demethylation, H3K9me2 could possibly generate a chromatin environment that protects the maternal chromosomes against demethylation. Other histone modifications such as H3 hypoacetylation may also contribute to this mechanism. This speculation is supported by the work of Spinaci et al who observed a positive influence of histone hyperacetylation on demethylation of maternal DNA in the mouse zygote [36].

H3K4me3 is found to be strongly associated with both maternal and paternal genomes in mouse, bovine and rabbit zygotes (Figure 1C). This modification is attributed to the transcriptionally active chromatin. It is known that in the mouse embryonic genome activation (EGA) starts in late zygotes and reaches its maximum at the two-cell stage [37]. The accumulation of H3K4me3 in late mouse zygotes may indicate the onset of transcriptional activity. The identical patterns in bovine and rabbit zygotes do not support this interpretation, since the EGA in these species does not occur before the eight- to 16-cell stage [37].

**Figure 9:**
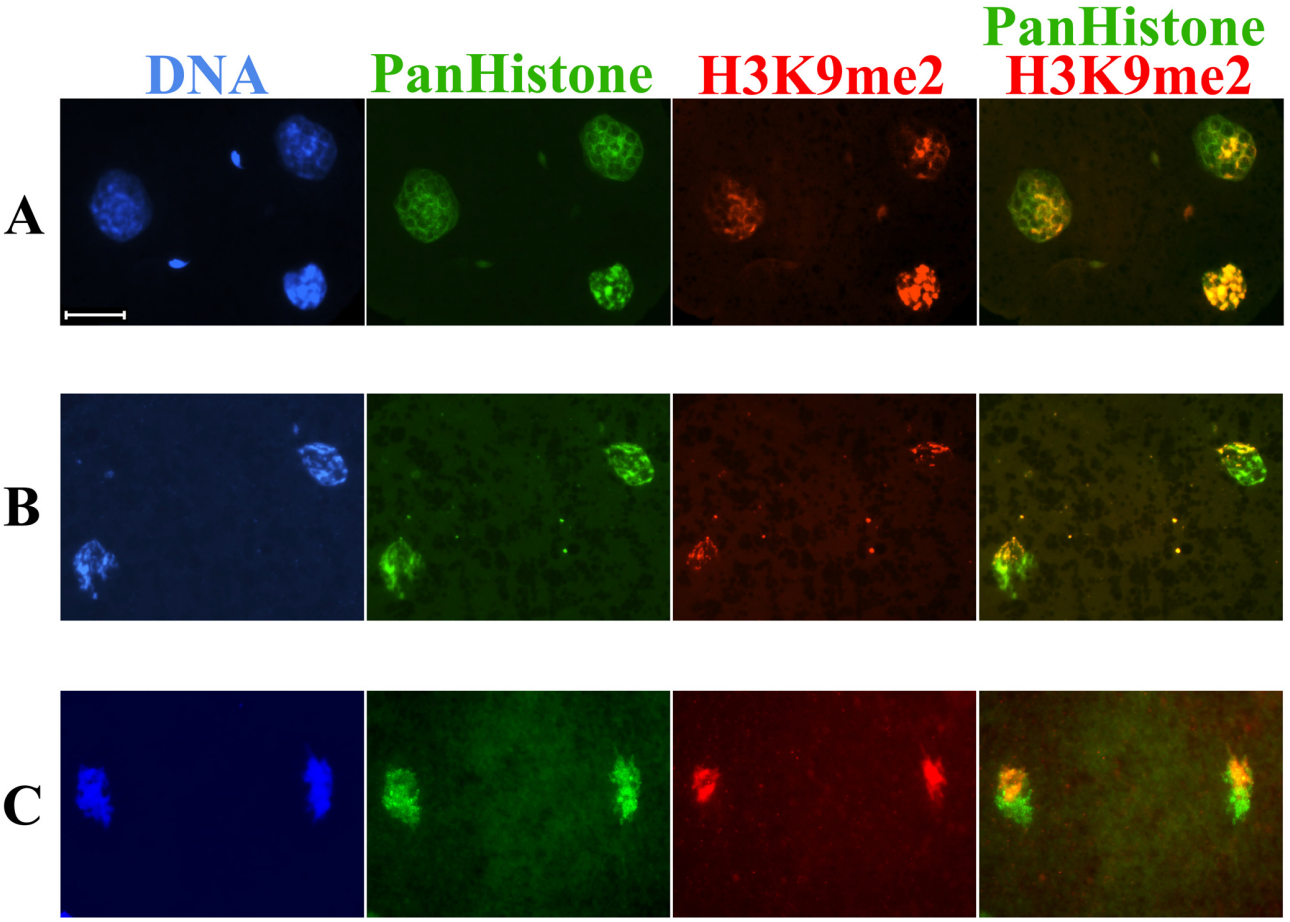
**H3K9me2 distribution in mouse (A), bovine (B) and rabbit (C) early two-cell embryos**. H3K9me2 was visualized using a specific rabbit polyclonal antibody. Core histones were visualized by a mouse monoclonal anti-Pan histone antibody. DNA was counterstained with DAPI. In all presented cases H3K9me2 compartmentalized asymmetrically in the nuclei of early two-cell embryos (*n *> 5). Scale bar 20 μm.

The different epigenetic signatures of paternal and maternal genomes established in zygotic pronuclei are maintained for at least one to two further cell divisions. Using epigenetic profiles such as DNA methylation and H3K9me2 as 'markers', spatially separated chromosomes can be observed in mouse two- and four-cell preimplantation embryos [28,38]. Indeed, using an H3K9me2-specific antibody we observed a similar spatial compartmentalization in bovine and rabbit early two-cell embryos (Figure 9). Overall, the pronuclear distributions and dynamics of the analyzed histone modifications are rather similar between mouse, rabbit and bovine zygotes.

## Conclusion

Our analysis shows that the rabbit oocyte possesses a DNA-demethylation activity. In rabbit zygotes the paternal chromosomes are significantly demethylated at advanced pronuclear stages. We also show that the dynamics of chromatin modifications are conserved in zygotes of mice, cattle and rabbit. Together these findings allow us to conclude that the basic mechanisms of epigenetic reprogramming are conserved in zygotes of these three mammalian species.

## Methods

### Animals

All animal experiments were carried out according to the German Animal Welfare law in agreement with the authorizing committee.

### *In vitro *fertilization and culture of mouse oocytes

(C57BL/6 × CBA)F1 adult mice were used as donors of sperm and oocytes. Mature oocytes were collected from superovulated females 14 hours post human chorionic gonadotropin (hCG) (Sigma-Aldrich, Schnelldorf, Germany) injection according to standard procedure [39]. Sperm isolation and *in vitro *fertilization (IVF) procedures were performed as described by Nagy [39]. Briefly, sperm was isolated from cauda epididymis of donor males and capacitated in pre-gassed KSOM medium for 1.5 hours. Isolated oocytes in cumulus cell mass were placed into 400 μl drops of modified KSOM medium with capacitated sperm and incubated in a CO_2 _incubator in a humidified atmosphere composed of 5% CO_2 _in air at 37°C for up to 20 hours.

### *In vitro *production of bovine embryos

Bovine embryos were produced as described [40]. Briefly, cumulus oocyte complexes (COCs) were isolated from ovaries obtained from a local slaughterhouse. *In vitro *maturation of oocytes was performed in TCM 199 medium containing 10 IU eCG and 5 IU hCG (Suigonan; Intervet, Tönisvorst, Germany) and 0.1% BSA fatty acid-free (Sigma-Aldrich, Schnelldorf, Germany) in a humidified atmosphere composed of 5% CO_2 _in air at 39°C for 24 hours. Following *in vitro *maturation, COCs were rinsed in fertilization medium (Fert-TALP supplemented with 6 mg/ml BSA) and fertilized in Fert-TALP containing 10 μM hypotaurine (Sigma Chemical Co., St. Louis, MO), 1 μM epinephrine (Sigma Chemical Co., St. Louis, MO), 0.1 IU/ml heparin (Serva, Germany) and 6 mg/ml BSA (Fraction V, Sigma-Aldrich, Schnelldorf, Germany). Frozen semen from one bull with proven fertility in IVF was used. The final sperm concentration added per fertilization drop was 1 × 10^6 ^sperm/ml. Fertilization was initiated during a 19-hour co-incubation under the same temperature and gas conditions as described for maturation. For *in vitro *culture, synthetic oviductal fluid (SOF) medium was employed supplemented with 4 mg/ml fatty acid-free BSA (Sigma-Aldrich, Schnelldorf, Germany).

### Obtaining rabbit zygotes and preimplantation stage embryos

Rabbit embryos were collected from superovulated outbred Zika rabbits mated with Zika males as described previously [19].

### Rabbit nuclear transfer

#### Recipient oocytes

Oocytes were obtained from sexually mature Zika breed rabbits. All experiments were carried out during the natural breeding season. Female rabbits were superovulated by injection of 100 IU pregnant mares' serum (Sigma-Aldrich, Schnelldorf, Germany) intramuscularly and 100 IU hCG (Sigma-Aldrich, Schnelldorf, Germany) intravenously 72 hours later. Mature oocytes were flushed from the oviducts 15 to 16 hours post-hCG injection in warm PBS supplemented with 4 mg/ml BSA. Cumulus cells were removed by gentle pipetting with a small-bore pipette after treatment of oocytes in 5 mg/ml hyaluronidase prepared in M199 (M199 medium containing 10% FCS) for 15 min at 38.5°C.

To induce the MII metaphase protrusion cumulus-free oocytes were treated with 0.6 μg/ml demecolcine (Sigma) in M199 for 40 min to 2 hours [41]. The extruded MII metaphase with a small amount of associated material was removed in M199 supplemented with 7.5 μg/ml cytochalasin B (Sigma-Aldrich, Schnelldorf, Germany) and 0.6 μg/ml demecolcine. Enucleated recipient oocytes were kept in M199.

#### Preparation of donor somatic cells

Rabbit fetal fibroblast (RFF) cells were isolated from individual fetuses at 15 to 16 days post-coitum, and cultured in DMEM in a humidified atmosphere of 5% CO_2 _in air at 38.5°C. RFFs from passage 1 to 8 were used as nuclear donors. Prior to nuclear transfer, attached cells were trypsinized, washed and suspended in M199. Since donor cells were cultured until confluency, the majority of cells were in G0/G1.

#### Nuclear transfer, fusion, activation and embryo culture

Transfer of donor karyoplast was carried out essentially as described previously [42]. Briefly, an individual nuclear donor cell was introduced under the zona pellucida of an enucleated oocyte in M199. Reconstructed embryos were produced after fusion of karyoplast-cytoplast complexes in Eppendorf fusion medium (Hamburg, Germany) with an Eppendorf Multiporator (Eppendorf, Hamburg, Germany) using a double electric pulse of 1.95 KV/cm for 25 μs. After 20 to 40 min incubation in M199, fused embryos were activated by the same electric pulse, then immediately incubated for one hour in 1.9 mM 6-dimethylaminopurine (6-DMAP) and 5.0 μg/ml CB prepared in Menezo B2 medium (INRA, Paris, France) containing 10% FCS in a humidified atmosphere of 5% CO_2 _in air at 38.5°C. After activation, cloned embryos were cultured in Menezo B2 medium until the desired stages were reached.

### Immunofluorescence staining

After removal of the zona pellucida by treatment with Acidic Tyrode's solution (Sigma-Aldrich, Schnelldorf, Germany), fertilized oocytes or cleavage-stage embryos were fixed for 20 min in 3.7% paraformaldehyde in PBS, and permeabilized with 0.2% Triton X-100 in PBS for 10 min at room temperature. The fixed zygotes were blocked overnight at 4°C in 1% BSA, 0.1% Triton X-100 in PBS. After blocking the embryos were incubated in the same solution with either anti PanHistone (mouse monoclonal, Roche), anti-H3K4me3 (rabbit polyclonal, Abcam, UK) or anti-H3K9me2 (rabbit polyclonal, a gift from T. Jenuwein [43]) antibodies at 4°C overnight, followed by several washes and incubation for 1 hour with anti-mouse secondary antibodies coupled with fluorescein (Sigma Chemical Co., St. Louis, MO), and anti-rabbit secondary antibodies coupled with Rhodamine Red-X (Jackson ImmunoResearch Laboratories Inc., USA). For α-5meC immunostaining, following the permeabilization step the embryos were placed into a drop of 4 M HCl for 10 in at room temperature to depurinate and thus to denature the DNA. The denaturation was stopped by neutralization in 100 mM TrisCl pH 8.0 for 5 min. Further blocking and immunostaining steps were as described above. Mouse monoclonal (Eurogentec, Belgium) or rabbit polyclonal (Megabase Research, USA) α-5meC antibodies were used. After final washes the embryos were placed on cleaned microscopy slides and mounted with a small drop of Vectashield (VectorLab, USA) mounting medium containing 0.5 μg/ml 4,6-diamino-2-phenylindole (DAPI) or 2 μg/ml propidium iodide (Sigma-Aldrich, Schnelldorf, Germany).

### Bisulfite treatment

We essentially followed the method described by Tierling et al [44] with minor modifications. Fifty rabbit one-cell NT embryos were washed in PBS, taken up in 1 to 2 μl and mixed with 12 μl of 2% molten low melting point agarose (in water) to form agarose beads for the bisulfite reaction. All individual sequences of clones, derived after cloning of bisulfite PCR product, were aligned and proved to be not clonal, which means they contained polymorphic sites and did not originate from the same DNA target molecule.

### PCR amplification from bisulfite-treated DNA, cloning, sequencing and COBRA analysis

PCR amplifications from bisulfite-treated DNA were performed according to [44]. Primers used for rabbit L1Oc7(8) variants of LINE1 repeated element [34] were:

5'-GTG TTT TAA GGG GAG TGT ATG TT-3' and 5'-AAA CCT TAC AAC CTT ATA ACC TTT AC-3'.

Primers used for *Rsat IIE *satellite amplification [45] were: 5'-ATA GTT GTT GGT TTG TTT TAA TTT TGG T-3' and 5'-GGT TAT TTC TTT TCT TTC ATA ATA AAT CTA-3'.

Annealing temperature for both reactions was 55°C and the number of cycles applied was 45. The resulting PCR products were cloned into pGEM-T vector (Promega, USA) and 12 individual clones were sequenced per experiment. For COBRA, PCR products of 32 single clones were amplified using plasmid-specific primers (M13 primers) and the resulting 'colony' PCR products were digested with T*aq*I restriction endonuclease (Fermentas, Germany). T*aq*I restriction sites are present at CpG positions # 3 and #6 of the L1Oc7(8) bisulfite sequence profiles and at CpG position #4 of the *Rsat IIE *sequence profiles.

### Immunofluorescence microscopy

The slides were analyzed on a Zeiss Axiovert 200 M inverted microscope equipped with the fluorescence module and B/W digital camera for imaging. The images were captured, pseudocolored and merged using AxioVision 4.4 software (Zeiss, Jena, Germany).

### Quantification of immunofluorescence data

The analysis was performed using ImageJ software. After the background subtraction the areas occupied by paternal or maternal pronuclei were defined manually and the integrated fluorescence signal densities (pixels) were calculated by the software. The relative amount of paternal methylation was calculated as the ratio of paternal to maternal pronucleus integrated fluorescence signal densities. The values obtained were then corrected by dividing them by the ratios (maternal/paternal) of the corresponding DNA signal intensities. Only zygotes with minimal or no overlap between paternal and maternal pronuclei were used for the analysis.

## Competing interests

The authors declare that they have no competing interests.

## Authors' contributions

KL conducted the experimental part of the work on mouse embryos, performed the immunostaining and DNA bisulfite conversion procedures, data acquisition, and analysis and co-wrote the manuscript. VZ, RH and FY conducted experiments on *in vivo *and somatic cell nuclear transfer-derived rabbit embryos. CW conducted experiments on bovine embryos. HN, EW, VZ and JW coordinated the work and co-wrote the manuscript. All authors read and approved the final manuscript.
